# Modulation of Mast Cell Reactivity by Lipids: The Neglected Side of Allergic Diseases

**DOI:** 10.3389/fimmu.2019.01174

**Published:** 2019-05-29

**Authors:** Philipp M. Hagemann, Stephanie Nsiah-Dosu, Jennifer Elisabeth Hundt, Karin Hartmann, Zane Orinska

**Affiliations:** ^1^Division of Experimental Pneumology, Research Center Borstel, Leibniz Lungenzentrum, Airway Research Center North, German Center for Lung Research (DZL), Borstel, Germany; ^2^Department of Dermatology, University of Luebeck, Luebeck, Germany; ^3^Luebeck Institute of Experimental Dermatology, University of Luebeck, Luebeck, Germany; ^4^Division of Allergy, Department of Dermatology, University of Basel, Basel, Switzerland

**Keywords:** mast cells, degranulation, allergy, lipids, lipid mediators, flippases, floppases, scramblases

## Abstract

Mast cells (MCs) have long been mainly regarded as effector cells in IgE-associated allergic disorders with potential immunoregulatory roles. Located close to the allergen entry sites in the skin and mucosa, MCs can capture foreign substances such as allergens, toxins, or noxious substances and are exposed to the danger signals produced by epithelial cells. MC reactivity shaped by tissue-specific factors is crucial for allergic responses ranging from local skin reactions to anaphylactic shock. Development of Th2 response leading to allergen-specific IgE production is a prerequisite for MC sensitization and induction of FcεRI-mediated MC degranulation. Up to now, IgE production has been mainly associated with proteins, whereas lipids present in plant pollen grains, mite fecal particles, insect venoms, or food have been largely overlooked regarding their immunostimulatory and immunomodulatory properties. Recent studies, however, have now demonstrated that lipids affect the sensitization process by modulating innate immune responses of epithelial cells, dendritic cells, and NK-T cells and thus crucially contribute to the outcome of sensitization. Whether and how lipids affect also MC effector functions in allergic reactions has not yet been fully clarified. Here, we discuss how lipids can affect MC responses in the context of allergic inflammation. Direct effects of immunomodulatory lipids on MC degranulation, changes in local lipid composition induced by allergens themselves and changes in lipid transport affecting MC reactivity are possible mechanisms by which the function of MC might be modulated.

## Introduction

Mast cells are long-living tissue-resident hematopoietic cells equipped with secretory granules containing a broad spectrum of biologically active mediators such as histamine, proteases, and cytokines (1, 2). Preferentially located in the skin and mucosa, MCs detect potentially dangerous or noxious substances in concert with danger signals produced by epithelial cells at damaged barriers. Extensive MC degranulation as an urgent response to different types of stimulation and its wide-ranging local or systemic effects are the reasons why MCs are the main effector cells in allergies (3). Here, we summarize recent findings describing how reactivity of MCs can be modulated by lipids and discuss how interference with intracellular lipid transport could affect MC reactivity.

## Lipid Structure and Molecular Features

Lipids are overall hydrophobic or amphipathic molecules consisting of a hydrophilic head group and a hydrophobic tail group connected either by esters or ether bonds. Other lipids like sterols consist of a ring structure with various modifications. Lipids, in contrast to proteins and nucleic acids, are synthesized by a series of specific interlinked enzymatic reactions generating a high diversity of different lipid molecules. According to their hydrophobic characteristics and chemically functional backbones, lipids are categorized into eight main groups, namely fatty acyls, glycerolipids, glycerophospholipids, sphingolipids, sterol lipids, prenol lipids, saccharolipids, and polyketides (http://www.lipidmaps.org). Lipids are essential in storage of energy, arrangement of signaling complexes, participation in signal processing as second messengers and building of membranes as physical barriers. Membranes in mammalian cells consist mainly of sphingolipids, glycerophospholipids, and cholesterol (4). They are fluidic bilayers characterized by different lipid compositions in their inner and outer sides where lipids together with proteins form highly ordered structures essential for organization of cellular compartments. An integral part of membranes is cholesterol. It is synthesized in the endoplasmic reticulum (ER), transported then to the Golgi complex, and further to the plasma membrane which shows the highest cholesterol concentration. Together with sphingolipids, cholesterol regulates the membrane permeability and facilitates organization of ordered protein islets. Glycerophospholipids and sphingolipids are also synthesized in the ER and further modified in the Golgi complex as well as the mitochondria (5–7). The lysosome, on the other hand, plays a crucial role in lipid sorting and metabolism (8). Enzymatically induced changes in the lipid composition of the membrane are associated with a new ordering of membrane proteins and altered membrane microdomains (9). In general, any change in the lipid or protein compartment of a membrane affects both partners and is therefore tightly controlled by the cell. MCs in particular undergo dramatic membrane reorganization while degranulation and recovery. The schematic structure of the different lipid categories and exemplary representatives of these categories with effects on MC functions are summarized in Table 1.

**Table 1:** Lipid categories and examples of lipids affecting MC reactivity.

**Lipid category**	**Schematic structure**	**Examples of lipids with effects on MC reactivity**	**References**
Fatty acyls	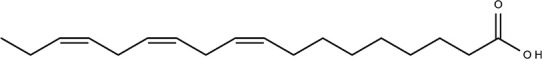 α-Linolenic acid	Fatty acids, Omega 3, and 6 polyunsaturated fatty acids PG, TX, LT, LX AEA	(10–13) (14–16) (17)
Glycerolipids	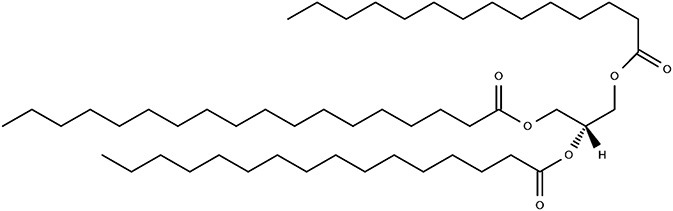 Triglyceride	2-AG	(17)
Glycerophospholipids	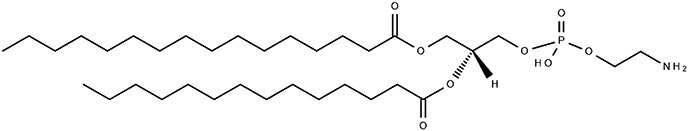 Phosphatidylethanolamine	PC, PE, PI, PS, PAF	(18–20)
Sphingolipids	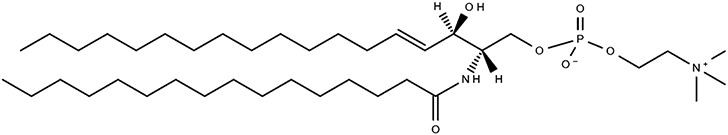 Sphingomyelin	Ceramide C1P, S1P	(21) (22, 23)
Sterol lipids	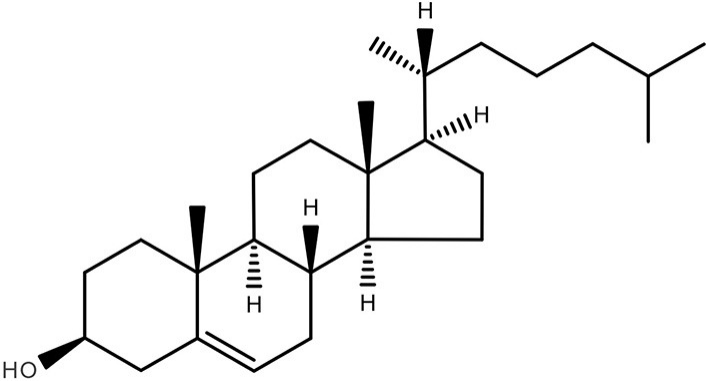 Cholesterol	Cholesterol Steroids Vitamin D_3_	(24–27) (28) (29, 30)
Prenol lipids	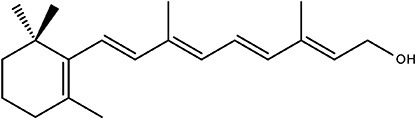 Vitamin A	Carotenoids Vitamin E	(11) (31)
Saccharolipds	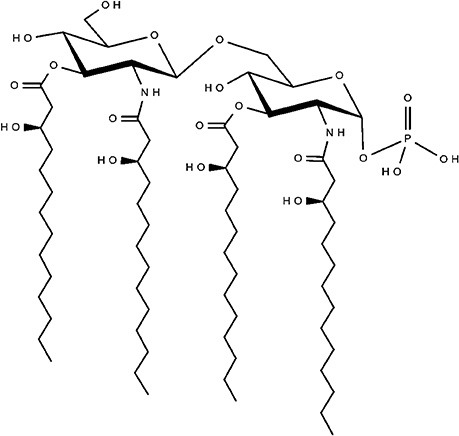 Lipid A -disaccharide-1-phosphate	LPS	(32, 33)
Polyketides	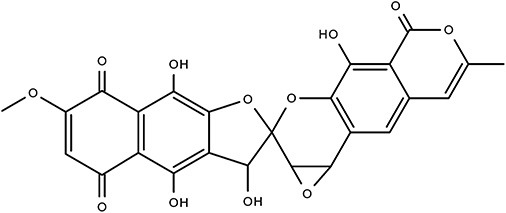 Griseorhodin A	Antibiotics Flavonoides	(34) (11)

*http://www.lipidmaps.org is a source for structure of lipids. TX, thromboxane; PI, phosphatidylinositol*.

## Production of Lipid Mediators is Associated With Changes in Mast Cell Reactivity

Activated in settings of allergic responses, mainly through stimulation of the high affinity receptor FcεRI complex by IgE-recognizing specific antigens, MCs release pre-stored biogenic amines, proteases, proteoglycans, chemokines as well as cytokines. In addition, MCs are well-known producers of different lipid mediators such as leukotrienes (LT) and prostaglandins (PG) (14, 20, 35) and production of these lipid mediators has in turn been shown to regulate MC functions. For example, MC-produced lipid mediators have been found to enhance inflammation in specific situations (36, 37) or to limit inflammation in other circumstances associated with reestablishment of tissue homeostasis (38, 39). Recent studies also demonstrated that enzymes responsible for production of lipid mediators belong to the MC-specific gene expression signature (40). Furthermore, *in vitro* generated connective tissue-like MCs and mucosal-like MCs differ in their eicosanoid patterns (41) and skin MCs are unique in showing the lowest expression levels of *Alox5* gene encoding 5-lipoxygenase (40), indicating that lipid mediator production is coordinated by tissue-specific regulatory mechanisms. Production of eicosanoid mediators, sphingolipid metabolites, and platelet-activating factor (PAF) by MCs is extensively reviewed elsewhere (15, 42). Thus, during allergic responses, MCs produce a variety of lipid mediators acting in a paracrine and autocrine manner. In addition, MC reactivity is modulated by lipid mediators produced by other cells exposed to environmental challenges.

## Endocannabinoids Affect Mast Cell Reactivity

Often overlooked regarding their modulatory effects on MC function are endocannabinoids-a group of bioactive lipids serving as secondary immune modulators participating in down-regulation of inflammatory processes (17, 43). The best characterized members of endocannabinoid lipid mediators are N-arachidonoylethanolamine (anandamide, AEA) and 2-arachidonoylglycerol (2-AG) (44, 45), which are derived from membrane phospholipids in response to physiological or pathological stimuli. Furthermore, new signaling mechanisms for intracellular transport and storage of endocannabinoids have been described (46–49). Endocannabinoids act through type-1 (CB1) and type-2 (CB2) G protein-coupled cannabinoid receptors, G protein-coupled receptor GPR55, transient receptor potential channel of the vanilloid subfamily 1 (TRPV1), and peroxisome proliferator-activated receptor γ (PPAR γ) (50). CB1 and CB2 are expressed on MCs (51) and initiate a series of signal transduction events that converge at the transcriptional level to regulate cell migration and production of cytokines and chemokines (52, 53). Acting in concert with GPR55, CB2 mediates signals inhibiting MC degranulation and cytokine synthesis (54). Described anti-fibrotic effects of cannabinoid receptors in different MC-related disease models (55, 56) together with the recently deciphered crystal structure of CB1 and CB2 (57, 58) will allow development of selective agonists and their implementation in novel therapeutic concepts for allergic diseases.

## Allergy-Associated Immunomodulatory Lipids act on Mast Cells

**Figure 1:**
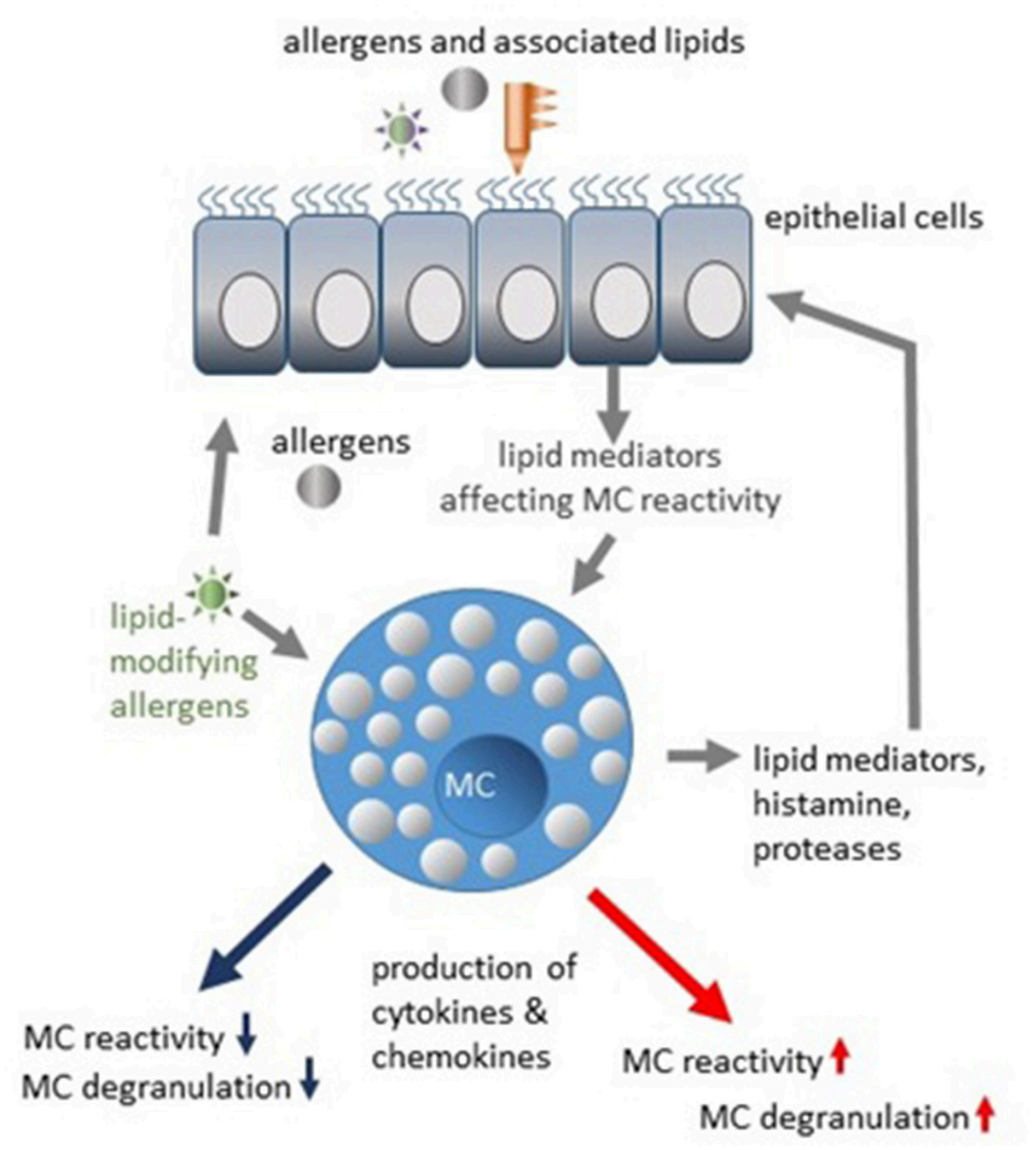
Modulation of MC reactivity by allergen-associated lipids and lipid mediators.

One would expect that allergen-associated lipids of plants or bacterial origin preferentially affect epithelial cells. Interaction of lipids with MCs might rather be possible in tissues with a damaged barrier (mainly by proteolytic activity of allergens) or indirectly in individuals showing previous sensitization and presence of specific IgE and active transport of IgE-antigen complexes containing lipids. MCs are regulated by lipids associated with different allergens, as extensively reviewed elsewhere (59, 60). Interestingly, epithelial cells from healthy donors sense allergens differently than epithelial cells from allergic patients (61). Therefore, enhanced reactivity of MCs could be the result of a combined action of allergen-lipid complexes and pro-allergic inflammatory mediators produced by epithelial cells (Figure 1). Degranulation of human lung MCs has been shown to be inhibited in coculture with bronchial epithelial cells (62), substantiating the hypothesis that epithelial cells can provide the inhibitory signals to MCs as well. Attractive candidates potentially limiting MC-reactivity are specialized pro-resolving mediators (SPMs) crucial for the resolution of inflammatory processes (63, 64). Four classes of SPMs have been characterized so far. Lipoxins (LX) are biosynthetic products of arachidonic acid. Resolvins, protectins, and maresins are products of eicosapentaenoic acid (EPA), docosapentaenoic acid (n-3DPA) or docosahexaenoic acid (DHA) (64). The epithelial cell-derived resolvins D1, D2, and lipoxin A4 have been found to suppress IgE-mediated histamine release from MCs via G-protein-coupled receptors (65). Furthermore, airway inflammation, mucus production, and airway hyperresponsiveness *in vivo* as well as MC degranulation and cytokine release were decreased by lipoxin B4 application (16), indicating therapeutic potential of pro-resolving lipid mediators in regulation of MC reactivity. Whether SPMs could be produced by MCs themselves, is unknown.

One major class of allergen-containing particles represent plant pollens, where pollen grains are coated with different lipids essential for plant fertilization (66). By interacting with immune cells and epithelial cells, pollen lipids may play an important role in immunoregulation. Two classes of pollen-associated lipid mediators (PALMs) have been described so far, namely LTB4-like mediators, which are monoxydroxylated derivatives of linoleic acid, and phytoprostanes generated from α-linolenic acid in response to oxidative stress (67). Effects of PALMs on MC degranulation have been reported for aqueous pollen extracts (APE) derived from birch pollen and for APE from *Ambrosia artemisiifolia* (68). Here, degranulation was induced in skin MCs of C57BL/6 mice by intradermal injection of APE in the absence of specific IgE. It is not known whether MC degranulation can be induced by APE themselves in the presence of an intact skin barrier. In experiments with RBL cells, it has been shown that *Ambrosia* pollen extract induces histamine release by a ROS-dependent mechanisms, but not β-hexosaminidase release (69). In experiments with mountain ceder (*Juniperus ashei*) pollen extract, release of both serotonin and β-hexosaminidase was induced in RBL cells in an IgE-independent, but ROS-dependent manner. Added to suboptimal IgE/AG concentrations, pollen extracts enhanced degranulation of RBL cells (70), although lipid components in particular extracts were not analyzed. Interestingly, persistent contact with grass pollen in early childhood has been found to represent one of various allergy-protective factors (71). However, whether lipids are essential for tolerance induction and whether MCs are directly involved in tolerance development remains to be investigated.

## Allergens Induce Changes in Mast Cell Lipid Composition

Interaction of honeybee venom phospholipase A2 (PLA2) with membrane lipids is an example how allergen-induced modification of lipids could tune MC reactivity. Insect venom, particularly *Hymenoptera* venoms, induces a pronounced Th2 response by coopting evolutionary conserved immunological and neurological mechanisms (3, 72). A mixture of different substances, including enzymes, toxic peptides, lipids, and biogenic amines, is transported into the skin by the insect sting and induces a local inflammatory reaction, leading to sensitization and IgE production. Phospholipase A2 is one of the two major honeybee (*Apis mellifera*) venom allergens (73, 74). Cleaving cell membrane phosphodiacylglycerides, PLA2 induces the release of lysophospholipids, particularly lysophosphatidylcholine (LPC), together with fatty acids. This local lipid remodeling can affect MC reactivity *per se* and lead to MC degranulation (75, 76) (Figure 1). The stimulatory effect on MCs is absent if the enzymatically inactive form of PLA2 is used (77). PLA2 enzymatic activity is also required to induce a Th2 response (78). Generated neoantigens in the skin are presented by the CD1a molecules of antigen-presenting cells (APCs) and then induce a polyclonal T cell response (79). Interestingly, the stimulatory effects of PLA2 were observed only in the presence of lipids, either venom- or host-derived, indicating that lipid and protein components act in concert to induce a T cell response (79). PLA2 activity has also been detected in house dust mite extracts (80), indicating that generation of lysophospholipids could be a part of allergic sensitization program. Moreover, an interaction between MCs and dendritic cells (DCs) has been demonstrated in contact hypersensitivity models (81, 82), where MCs were “cross-dressed” with DC MHC class II complexes. How the local changes of lipid composition, induced by e.g., PLA2 activity, modulate the MC-DC interaction and which functional consequences this would have for the T cell response in allergic settings remains to be further elucidated.

## Membrane Lipid Organizing Enzymes are Targets to Modulate Mast Cell Activation

Organization of membrane lipids plays an important role in regulation of MC degranulation. The inner leaflet, facing the inside of the cell, contains negatively charged amino-phospholipids, and phosphatidylethanolamine (PE). The outer leaflet, facing the outside environment, contains phosphatidylcholine (PC) and sphingomyelin. Asymmetric distribution of phospholipids in the plasma membrane plays an essential role in regulation of MC exocytosis (83). Interestingly, one of the earliest events in MC degranulation is a redistribution of phosphatidylinositol 4,5-bisphospate [PtdIns(4,5)*P2*] disappearing from the plasma membrane within seconds after stimulation (84). Furthermore, MC degranulation is associated with reversible phosphatidylserine (PS) translocation to the plasma membrane (85), in contrast to various other cell types, in which the PS translocation represents an apoptotic “eat-me” signal. PS exposure can implicate endocytosis, acquisition of membrane curvature, regulation of transmembrane proteins, interactions with cytoskeletal elements as well as involvement in PS signaling (19). Lipid transporting phospholipid scramblase 1 (PLSCR1), floppase ABCA1 or transmembrane protein TMEM16F are the candidates responsible for PS translocation (19, 86). Interestingly, PS translocation in MCs could be induced not only by FcεRI-mediated activation. Crosslinking of glycosylphosphatidylinositol-anchored proteins by specific antibodies or lectins also induce PS externalization, using probably a different Ca^2+^-independent mechanism (87). It seems that the context in which MCs recognize PS is important, since free PS and lyso-PS enhance FcεRI-mediated degranulation (88) and phosphatidylserine-specific phospholipase A1, released e.g., by activated platelets, generates lyso-PS and strongly enhances MC histamine release (75). However, recognition of PS on the surface of apoptotic cells by the inhibitory receptor CD300a leads to a downregulation of inflammatory cytokine and chemokine production (89). Also, rodent MCs express α-galactosyl derivatives of the ganglioside GD1b (90). Antibodies recognizing this ganglioside inhibit degranulation and histamine release by modulating FcεRI endocytosis (90, 91) but in contrast, are also able to promote release of cytokines and lipid mediators (92).

**Figure 2:**
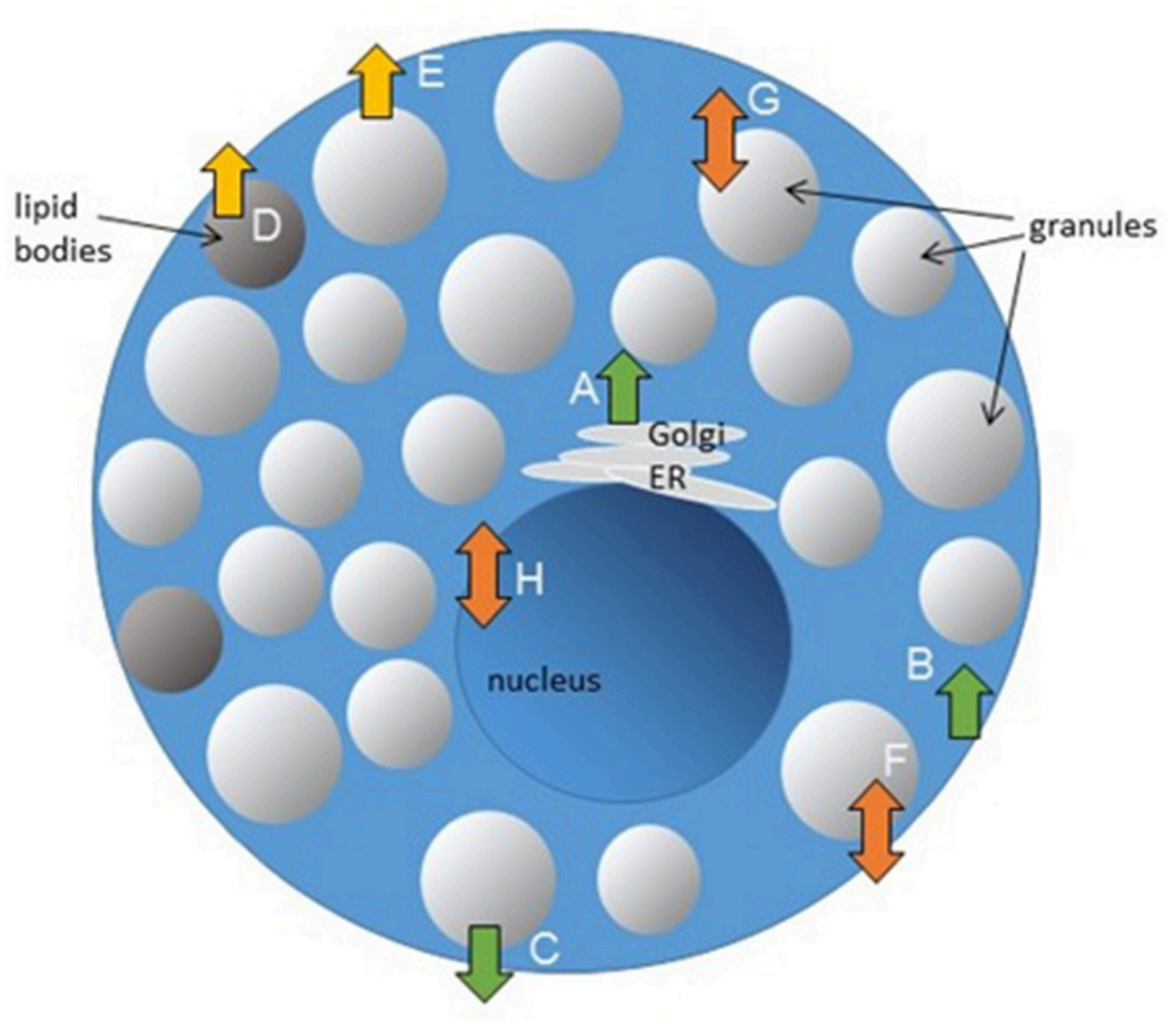
Potential effects of flippases, floppases, and scramblases on MC function. Lipid transporting enzymes could be involved in regulation of different cellular processes. Granule biogenesis **(A)**, endocytosis **(B)**, and exocytosis **(C)** could be regulated by flippases. Floppases are participating in transport of lipid mediators **(D)** and could be involved in regulation of MC exocytosis **(E)**. Scramblases regulate FcεRI-mediated signaling and MC degranulation **(F)**, could be potentially involved in TLR-signaling in endosomes **(G)** and regulation of gene transcription **(H)**.

Lipid content and distribution in membranes are regulated by different enzymes. Three types of phospholipid transportation enzymes are responsible for maintenance of the phospholipid asymmetry in membranes: (1) flippases that catalyze translocations of phospholipids between membrane leaflets in an energy-dependent or-independent manner, primarily from the external to the internal leaflet, (2) floppases that transport lipids from the cytoplasmic leaflet to the external membrane leaflet, and (3) scramblases that move lipids between the two leaflets [as reviewed in Pomorski and Menon (93)]. Potential effects of flippases, floppases, and scramblases on MC function are outlined in Figure 2.

Flippases are members of the P4-type ATPase family with a similar structure containing 10 transmembrane domains, an actuator domain, a phosphorylation domain, and a nucleotide-binding domain associating with an accessory subunit Cdc50, forming a heterodimeric complex. In mammals, 14 different P4-ATPases have been identified as heterodimers consisting of a catalytic subunit in association with one member of the Cdc50 family (94). Many P4-ATPases are ubiquitously expressed and have been implicated in different metabolic diseases (95). P4-ATPases are also involved in the phospholipid transport between different subcellular compartments and are responsible for maintenance of phospholipid asymmetry in different cell types. Lipid transport by P4-ATPases is lipid-specific, head group-, and backbone-dependent (96). P4-ATPases regulate vesicular trafficking and the bidirectional vesicular transport between the Golgi complex and early endosomes, but also vesicle biogenesis by enriching specific phospholipids in yeast cells, formation of post-Golgi vesicles in plant cells, as well as maintenance of membrane integrity and secretory processes (95, 97). Involvement of this class of lipid transporters in MC granule biogenesis and exocytosis is obvious. Genetic models with MC-specific P4-ATPase inactivation/overexpression could help to better understand essential regulatory steps in granule development, maturation and degranulation of MCs. Improvement of the knowledge on MC granule organization will also help to develop new strategies to interfere with MC degranulation.

Lipid transporters, shipping lipids from the inner membrane leaflet to the outer leaflet, are ATP-biding cassette (ABC) proteins, originally identified as multidrug resistance gene products in tumor cells. ABC proteins are encoded as single polypeptides, which can form homo- or heterodimers, contain an ATP-binding site, a nucleotide-binding domain and up to 17 transmembrane domains (98). Leukotriene C4 was the first lipid mediator described, transported by ABCC1/MRP1 (99). Different other lipid mediators such as prostaglandin A2 (PGA2) and 15-deoxy-Δ ^(12, 14)^ PGJ2, lysophosphatidylinositol (LPI) are also transported by specific ABC proteins (100, 101). In MCs with downregulated ABCC1/MRP1 expression, transport of S1P was strongly reduced, indicating an ABC-dependent regulation of MC chemotaxis and migration (102). How the lipid transport can be modulated by targeting other ABC proteins and how this will affect MC function is currently unknown. Mutations of ABC lipid transporters are responsible for several human diseases, such as neonatal surfactant deficiency (ABCA3 mutation) or Tangier disease (ABCA1 mutation), characterized by decreased removal of cholesterol from peripheral tissues (103).

Scramblases are structurally related proteins, containing a DNA-binding domain, a palmitoylation motif, a Ca^2+^-binding motif, transmembrane domains and a nuclear localization signal. Located in the plasma membrane, scramblases are involved in the Ca^2+^-dependent distribution of phospholipids (scrambling) (104). In 2008, the group of Benhamou identified phospholipid scramblase 1 (PLSCR1) as specific regulator of FcεRI signaling (105). Initially, PLSCR1 was only marked responsible for the rapid redistribution of phospholipids between two leaflets of the plasma membrane after cell activation or apoptosis, leading to the disruption of their asymmetric distribution (106–108). In the meantime, however, it is known that PLSCR1 serves numerous functions beyond the redistribution of phospholipids, such as the regulation of cell proliferation, differentiation, apoptosis, and tumor development (109–112). PLSCR1 requires palmitoylation to be stabilized at the plasma membrane. In the absence of palmitoylation, it is found in the nucleus, where it can bind DNA and activate the transcription of the inositol phosphate-3 (IP3) receptor (113, 114). When palmitoylated and localized at the plasma membrane, it participates in the epidermal growth factor signaling (115) by amplifying activation of the tyrosine kinase Src (116).

Knock-down of PLSCR1 in RBL-2H3 cells significantly impaired FcεRI-mediated degranulation and release of vascular endothelial growth factor (105). Earlier, Pastorelli had already observed that phosphorylation of PLSCR1 is increased following the engagement of FcεRI in RBL-2H3 cells (117). Tyrosine phosphorylation of PLSCR1 following FcεRI aggregation relies on Lyn and Syk tyrosine kinases and partially also on calcium mobilization. In contrast, Fyn signaling negatively regulated PLSCR1 phosphorylation, suggesting a complex modulation of FcεRI-dependent MC activation by PLSCR1 (105, 118). *In vivo* studies using *Plscr1*^−/−^ mice showed reduced FcεRI-dependent passive systemic anaphylaxis and serum histamine levels compared to wild-type mice (119), demonstrating the involvement of PLSCR1 in IgE-mediated anaphylaxis without affecting the phenotype or tissue distribution of resting MCs. Surprisingly, anaphylactic reactions induced by direct injection of histamine were slightly increased in *Plscr1*^−/−^ animals, indicating that PLSCR1 also counter-regulates IgE-dependent anaphylaxis at later stages.

The modulatory ability of PLSCR1, allowing increased as well as decreased biological responses, might serve to sophisticatedly regulate inflammation, host defense, tissue remodeling and homoeostasis and provide a rationale for exploiting PLSCR1 as therapeutic target in allergies (119, 120). Interestingly, in plasmacytoid DCs, PLSCR1 interacts with TLR9, and regulates the type I IFN response by modulating endosomal trafficking of TLR9 (121). Also, scramblase 2 (PLSCR2) has been found to be involved in the antiviral response. PLSCR2 binds to STAT3 and in this way also participates in downregulating the type I interferon response (122). Whether comparable effects will be observed in MCs and whether the antiviral response of MCs is compromised in the absence of scramblases remains an open question for future investigations. Mice deficient for scramblase 3 developed metabolic syndrome and lipid accumulation in abdominal fat pads (123).

Modulation of MC degranulation by affecting the lipid composition of the cell membrane or the enzyme activity modulating the lipid distribution of the membrane are potential emerging therapeutic strategies for the treatment of allergic diseases.

## Conclusion and Perspectives

The rapidly emerging field defining modulation of MC reactivity by lipids, in addition to proteins, reveals novel and unprecedented targets, which may serve to preclude MC effects in allergic reactions. Active substances secreted by MCs have already been studied extensively, but data on the overall lipid composition of MCs and on stimulus-specific as well as sub-cell type-specific lipidomic data are still missing. Direct effects of immunomodulatory lipids on MC degranulation, changes in MC lipid composition induced by allergens themselves and changes in lipid transport and metabolism in MCs have not yet been comprehensively investigated. Furthermore, current studies investigating MC lipids are often limited by use of non-physiologic conditions or narrow restriction of the lipids that were analyzed. Therefore, addition of modern lipidomic approaches to the toolbox of immunology and cell biology is crucial. Hereby, the added knowledge of lipid production and regulation together with deep understanding of MC biology will help find new mechanisms regulating MC responses. Coupled with this, an in-depth knowledge will be considerably advantageous for patients with anaphylaxis, asthma, allergic rhinitis, eczema, urticaria, mastocytosis, and other allergic diseases.

## Author Contributions

PH, SN-D, JH, KH, and ZO wrote sections of the manuscript. All authors contributed to manuscript revision, read, and approved the submitted version.

### Conflict of Interest Statement

The authors declare that the research was conducted in the absence of any commercial or financial relationships that could be construed as a potential conflict of interest. The handling Editor declared a shared affiliation, though no other collaboration, with the authors PH and ZO.

## References

[1] Mukai K Tsai M Saito H Galli SJ. Mast cells as sources of cytokines, chemokines, and growth factors. Immunol Rev. (2018) 282:121–50. 10.1111/imr.12634 29431212 PMC5813811

[2] Wernersson S Pejler G. Mast cell secretory granules: armed for battle. Nat Rev Immunol. (2014) 14:478–94. 10.1038/nri3690 24903914

[3] Palm NW Rosenstein RK Medzhitov R. Allergic host defences. Nature. (2012) 484:465–72. 10.1038/nature11047 22538607 PMC3596087

[4] Storck EM Özbalci C Eggert US. Lipid cell biology: a focus on lipids in cell division. Ann Rev Biochem. (2018) 87:839–69. 10.1146/annurev-biochem-062917-012448 29494237

[5] Litvinov DY Savushkin EV Dergunov AD. Intracellular and plasma membrane events in cholesterol transport and homeostasis. J Lipids. (2018) 2018:1–22. 10.1155/2018/3965054 30174957 PMC6106919

[6] van Meer G de Kroon AIPM. Lipid map of the mammalian cell. J Cell Sci. (2011) 124:5–8. 10.1242/jcs.071233 21172818

[7] van Meer G Voelker DR Feigenson GW. Membrane lipids: where they are and how they behave. Nat Rev Mol Cell Biol. (2008) 9:112–24. 10.1038/nrm2330 18216768 PMC2642958

[8] Thelen AM Zoncu R. Emerging roles for the lysosome in lipid metabolism. Trends Cell Biol. (2017) 27:833–50. 10.1016/j.tcb.2017.07.006 28838620 PMC5653458

[9] Márquez MG Favale NO Leocata Nieto F Pescio LG Sterin-Speziale N. Changes in membrane lipid composition cause alterations in epithelial cell–cell adhesion structures in renal papillary collecting duct cells. Biochim Biophy Acta. (2012) 1818:491–501. 10.1016/j.bbamem.2011.11.018 22155258

[10] Gueck T Seidel A Fuhrmann H. Effects of essential fatty acids on mediators of mast cells in culture. Prostagl Leukotr Essent Fatty Acids. (2003) 68:317–22. 10.1016/S0952-3278(03)00022-X 12711248

[11] Hagenlocher Y Lorentz A. Immunomodulation of mast cells by nutrients. Mol Immunol. (2015) 63:25–31. 10.1016/j.molimm.2013.12.005 24524883

[12] Ju H-R Wu H-Y Nishizono S Sakono M Ikeda I Sugano M. Effects of dietary fats and curcumin on IgE-mediated degranulation of intestinal mast cells in brown norway rats. Biosci Biotechnol Biochem. (1996) 60:1856–60. 10.1271/bbb.60.1856 8987863

[13] Wang X Kulka M. n-3 Polyunsaturated fatty acids and mast cell activation. J Leukocyte Biol. (2015) 97:859–71. 10.1189/jlb.2RU0814-388R 25765678

[14] Samuelsson B Dahlen S Lindgren J Rouzer C Serhan C. Leukotrienes and lipoxins: structures, biosynthesis, and biological effects. Science. (1987) 237:1171–6. 10.1126/science.2820055 2820055

[15] Boyce JA. Mast cells and eicosanoid mediators: a system of reciprocal paracrine and autocrine regulation. Immunol Rev. (2007) 217:168–85. 10.1111/j.1600-065X.2007.00512.x 17498059

[16] Karra L Haworth O Priluck R Levy BD Levi-Schaffer F Lipoxin B4 promotes the resolution of allergic inflammation in the upper and lower airways of mice. Mucosal Immunol. (2015) 8:852–62. 10.1038/mi.2014.116 25465102 PMC4454640

[17] Chiurchiù V Battistini L Maccarrone M. Endocannabinoid signalling in innate and adaptive immunity. Immunology. (2015) 144:352–64. 10.1111/imm.12441 25585882 PMC4557672

[18] Byrne R Larijani B The role of phosphoinositides in mast cell signalling. Signal Trans. (2006) 6:81–91. 10.1002/sita.200500074

[19] Shimoda LMN Dixon AM Speck M Stokes AJ Turner H Umemoto EY. Beyond apoptosis: the mechanism and function of phosphatidylserine asymmetry in the membrane of activating mast cells. BioArchitecture. (2014) 4:127–37. 10.1080/19490992.2014.995516 25759911 PMC4914033

[20] Schauberger E Peinhaupt M Cazares T Lindsley AW. Lipid mediators of allergic disease: pathways, treatments, and emerging therapeutic targets. Curr Allergy Asthma Rep. (2016) 16:48. 10.1007/s11882-016-0628-3 27333777 PMC5515624

[21] Chiba N Masuda A Yoshikai Y Matsuguchi T. Ceramide inhibits LPS-induced production of IL-5, IL-10, and IL-13 from mast cells. J Cell Physiol. (2007) 213:126–36. 10.1002/jcp.21101 17458900

[22] Olivera A Rivera J. Sphingolipids and the balancing of immune cell function: lessons from the mast cell. J Immunol. (2005) 174:1153–8. 10.4049/jimmunol.174.3.1153 15661867

[23] Sturgill JL. Sphingolipids and their enigmatic role in asthma. Adv Biol Regul. (2018) 70:74–81. 10.1016/j.jbior.2018.09.001 30197277 PMC6560640

[24] Baumruker T. Activation of mast cells by incorporation of cholesterol into rafts. Int Immunol. (2003) 15:1207–18. 10.1093/intimm/dxg120 13679390

[25] Kato N Nakanishi M Hirashima N. Cholesterol depletion inhibits store-operated calcium currents and exocytotic membrane fusion in RBL-2H3 cells. Biochemistry. (2003) 42:11808–14. 10.1021/bi034758h 14529292

[26] Surviladze Z Dráberová L Kovárová M Boubelík M Dráber P. Differential sensitivity to acute cholesterol lowering of activation mediated via the high-affinity IgE receptor and Thy-1 glycoprotein. Eur J Immunol. (2001) 31:1–10. 10.1002/1521-4141(200101)31:1&lt;1::AID-IMMU1&gt;3.0.CO;2-W 11169432

[27] Kovarova M Wassif CA Odom S Liao K Porter FD Rivera J. Cholesterol deficiency in a mouse model of Smith-Lemli-Opitz syndrome reveals increased mast cell responsiveness. J Exp Med. (2006) 203:1161–71. 10.1084/jem.20051701 16618793 PMC2121200

[28] Mori T Abe N Saito K Toyama H Endo Y Ejima Y. Hydrocortisone and dexamethasone dose-dependently stabilize mast cells derived from rat peritoneum. Pharmacol Rep. (2016) 68:1358–65. 10.1016/j.pharep.2016.09.005 27710865

[29] Liu Z-Q Li X-X Qiu S-Q Yu Y Li M-G Yang L-T. Vitamin D contributes to mast cell stabilization. Allergy. (2017) 72:1184–92. 10.1111/all.13110 27998003

[30] Yip K-H Kolesnikoff N Yu C Hauschild N Taing H Biggs L Mechanisms of vitamin D3 metabolite repression of IgE-dependent mast cell activation. J Allergy Clini Immunol. (2014) 133:1356–64.e14. 10.1016/j.jaci.2013.11.030 PMC4154631 24461581

[31] Zingg J. Vitamin E and mast cells. In: Lidwack G, editor. Vitamins and Hormones. Amsterdam: Elsevier. p. 393–418. 10.1016/S0083-6729(07)76015-6 17628183

[32] Masuda A Yoshikai Y Aiba K Matsuguchi T. Th2 cytokine production from mast cells is directly induced by lipopolysaccharide and distinctly regulated by c-Jun N-terminal kinase and p38 pathways. J Immunol. (2002) 169:3801–0. 10.4049/jimmunol.169.7.3801 12244175

[33] Varadaradjalou S Féger F Thieblemont N Hamouda NB Pleau J-M Dy M. Toll-like receptor 2 (TLR2) and TLR4 differentially activate human mast cells. Eur J Immunol. (2003) 33:899–906. 10.1002/eji.200323830 12672055

[34] Kazama I Saito K Baba A Mori T Abe N Endo Y. Clarithromycin dose-dependently stabilizes rat peritoneal mast cells. Chemotherapy. (2016) 61:295–303. 10.1159/000445023 27088971

[35] Kulinski JM Muñoz-Cano R Olivera A. Sphingosine-1-phosphate and other lipid mediators generated by mast cells as critical players in allergy and mast cell function. Eur J Pharmacol. (2016) 778:56–67. 10.1016/j.ejphar.2015.02.058 25941085 PMC4630215

[36] Bankova LG Lai J Yoshimoto E Boyce JA Austen KF Kanaoka Y. Leukotriene E_4_ elicits respiratory epithelial cell mucin release through the G-protein–coupled receptor, GPR99. Proc Natl Acad Sci USA. (2016) 113:6242–7. 10.1073/pnas.1605957113 27185938 PMC4896673

[37] Samuchiwal SK Boyce JA. Role of lipid mediators and control of lymphocyte responses in type 2 immunopathology. J Allergy Clini Immunol. (2018) 141:1182–90. 10.1016/j.jaci.2018.02.006 29477727

[38] Nakamura T Fujiwara Y Yamada R Fujii W Hamabata T Lee MY Mast cell–derived prostaglandin D 2 attenuates anaphylactic reactions in mice. J Allergy Clini Immunol. (2017) 140:630–2.e9. 10.1016/j.jaci.2017.02.030 28457595

[39] Shimanaka Y Kono N Taketomi Y Arita M Okayama Y Tanaka Y. Omega-3 fatty acid epoxides are autocrine mediators that control the magnitude of IgE-mediated mast cell activation. Nat Med. (2017) 23:1287–97. 10.1038/nm.4417 29035365

[40] The Immunological Genome Project Consortium Dwyer DF Barrett NA Austen KF. Expression profiling of constitutive mast cells reveals a unique identity within the immune system. Nat Immunol. (2016) 17:878–87. 10.1038/ni.3445 27135604 PMC5045264

[41] Lundström SL Saluja R Adner M Haeggström JZ Nilsson G Wheelock CE. Lipid mediator metabolic profiling demonstrates differences in eicosanoid patterns in two phenotypically distinct mast cell populations. J Lipid Res. (2013) 54:116–26. 10.1194/jlr.M030171 23034214 PMC3520518

[42] Olivera A Rivera J. An emerging role for the lipid mediator sphingosine-1-phosphate in mast cell effector function and allergic disease. Mast Cell Biol. (2011) 716:123–42. 10.1007/978-1-4419-9533-9_8 21713655 PMC3214605

[43] Migalovich-Sheikhet H Friedman S Mankuta D Levi-Schaffer F. Novel identified receptors on mast cells. Front Immunol. (2012) 3:238. 10.3389/fimmu.2012.00238 22876248 PMC3410575

[44] Devane W Hanus L Breuer A Pertwee R Stevenson L Griffin G. Isolation and structure of a brain constituent that binds to the cannabinoid receptor. Science. (1992) 258:1946–9. 10.1126/science.1470919 1470919

[45] Mechoulam R Ben-Shabat S Hanus L Ligumsky M Kaminski NE Schatz AR. Identification of an endogenous 2-monoglyceride, present in canine gut, that binds to cannabinoid receptors. Biochem Pharmacol. (1995) 50:83–90. 10.1016/0006-2952(95)00109-D 7605349

[46] Oddi S Fezza F Pasquariello N De Simone C Rapino C Dainese E. Evidence for the intracellular accumulation of anandamide in adiposomes. Cell Mol Life Sci. (2008) 65:840–50. 10.1007/s00018-008-7494-7 18213445 PMC11131627

[47] Oddi S Fezza F Pasquariello N D'Agostino A Catanzaro G De Simone C. Molecular identification of albumin and Hsp70 as cytosolic anandamide-binding proteins. Chem Biol. (2009) 16:624–32. 10.1016/j.chembiol.2009.05.004 19481477

[48] Kaczocha M Glaser ST Chae J Brown DA Deutsch DG. Lipid droplets are novel sites of *N*-acylethanolamine Inactivation by fatty acid amide hydrolase-2. J Biol Chem. (2010) 285:2796–806. 10.1074/jbc.M109.058461 19926788 PMC2807334

[49] Maccarrone M Dainese E Oddi S. Intracellular trafficking of anandamide: new concepts for signaling. Trends Biochem Sci. (2010) 35:601–8. 10.1016/j.tibs.2010.05.008 20570522

[50] Pertwee R. Receptors and channels targeted by synthetic cannabinoid receptor agonists and antagonists. Curr Med Chem. (2010) 17:1360–81. 10.2174/092986710790980050 20166927 PMC3013229

[51] Samson M-T Small-Howard A Shimoda LMN Koblan-Huberson M Stokes AJ Turner H. Differential roles of CB1 and CB2 cannabinoid receptors in mast cells. J Immunol. (2003) 170:4953–62. 10.4049/jimmunol.170.10.4953 12734338

[52] Sugawara K Zákány N Hundt T Emelianov V Tsuruta D Schäfer C. Cannabinoid receptor 1 controls human mucosal-type mast cell degranulation and maturation *in situ*. J Allergy Clini Immunol. (2013) 132:182–93.e8. 10.1016/j.jaci.2013.01.002 23453134

[53] Sugawara K Bíró T Tsuruta D Tóth BI Kromminga A Zákány N. Endocannabinoids limit excessive mast cell maturation and activation in human skin. J Allergy Clini Immunol. (2012) 129:726–38.e8. 10.1016/j.jaci.2011.11.009 22226549

[54] Cruz SL Sánchez-Miranda E Castillo-Arellano JI Cervantes-Villagrana RD Ibarra-Sánchez A González-Espinosa C. Anandamide inhibits FcεRI-dependent degranulation and cytokine synthesis in mast cells through CB2 and GPR55 receptor activation. Possible involvement of CB2-GPR55 heteromers. Int Immunopharm. (2018) 64:298–307. 10.1016/j.intimp.2018.09.006 30243065

[55] Zhou L Zhou S Yang P Tian Y Feng Z Xie X-Q. Targeted inhibition of the type 2 cannabinoid receptor is a novel approach to reduce renal fibrosis. Kidney Int. (2018) 94:756–72. 10.1016/j.kint.2018.05.023 30093080 PMC6151282

[56] Vuolo F Abreu SC Michels M Xisto DG Blanco NG Hallak JE. Cannabidiol reduces airway inflammation and fibrosis in experimental allergic asthma. Eur J Pharmacol. (2019) 843:251–9. 10.1016/j.ejphar.2018.11.029 30481497

[57] Krishna Kumar K Shalev-Benami M Robertson MJ Hu H Banister SD Hollingsworth SA. Structure of a signaling cannabinoid receptor 1-G protein complex. Cell. (2019) 176:448–58.e12. 10.1016/j.cell.2018.11.040 30639101 PMC6461403

[58] Li X Hua T Vemuri K Ho J-H Wu Y Wu L. Crystal structure of the human cannabinoid receptor CB2. Cell. (2019) 176:459–67.e13. 10.1016/j.cell.2018.12.011 30639103 PMC6713262

[59] Bublin M Eiwegger T Breiteneder H. Do lipids influence the allergic sensitization process? J Allergy Clini Immunol. (2014) 134:521–9. 10.1016/j.jaci.2014.04.015 24880633 PMC4151997

[60] del Moral MG Martínez-Naves E. The role of lipids in development of allergic responses. Imm Netw. (2017) 17:133. 10.4110/in.2017.17.3.133 28680374 PMC5484643

[61] Mattila P Renkonen J Toppila-Salmi S Parviainen V Joenväärä S Alff-Tuomala S. Time-series nasal epithelial transcriptomics during natural pollen exposure in healthy subjects and allergic patients. Allergy. (2010) 65:175–83. 10.1111/j.1398-9995.2009.02181.x 19804444

[62] Yang W Wardlaw AJ Bradding P. Attenuation of human lung mast cell degranulation by bronchial epithelium. Allergy. (2006) 61:569–75. 10.1111/j.1398-9995.2006.01041.x 16629786

[63] Basil MC Levy BD. Specialized pro-resolving mediators: endogenous regulators of infection and inflammation. Nat Rev Immunol. (2015) 16:51. 10.1038/nri.2015.4 26688348 PMC5242505

[64] Serhan CN Levy BD. Resolvins in inflammation: emergence of the pro-resolving superfamily of mediators. J Clin Invest. (2018) 128:2657–69. 10.1172/JCI97943 29757195 PMC6025982

[65] Martin N Ruddick A Arthur GK Wan H Woodman L Brightling CE. Primary human airway epithelial cell-dependent inhibition of human lung mast cell degranulation. PLoS ONE. (2012) 7:e43545. 10.1371/journal.pone.0043545 22970103 PMC3428358

[66] Bashir MEH Lui JH Palnivelu R Naclerio RM Preuss D. Pollen lipidomics: lipid profiling exposes a notable diversity in 22 allergenic pollen and potential biomarkers of the allergic immune response. PLoS ONE. (2013) 8:e57566. 10.1371/journal.pone.0057566 23469025 PMC3585183

[67] Gilles S Behrendt H Ring J Traidl-Hoffmann C. The pollen enigma: modulation of the allergic immune response by non-allergenic, pollen-derived compounds. Curr Pharm Design. (2012) 18:2314–9. 10.2174/138161212800166040 22390694

[68] Metz M Gilles S Geldmacher A Behrendt H Traidl-Hoffmann C Maurer M. Evidence for non-allergic mast cell activation in pollen-associated inflammation. J Invest Dermatol. (2011) 131:987–90. 10.1038/jid.2010.419 21248769

[69] Chodaczek G Bacsi A Dharajiya N Sur S Hazra TK Boldogh I. Ragweed pollen-mediated IgE-independent release of biogenic amines from mast cells via induction of mitochondrial dysfunction. Mol Immunol. (2009) 46:2505–14. 10.1016/j.molimm.2009.05.023 19501909 PMC2713802

[70] Endo S Hochman DJ Midoro-Horiuti T Goldblum RM Brooks EG. Mountain cedar pollen induces IgE-independent mast cell degranulation, IL-4 production, and intracellular reactive oxygen species generation. Cell Immunol. (2011) 271:488–95. 10.1016/j.cellimm.2011.08.019 21944563 PMC3195927

[71] Sudre B Vacheyrou M Braun-Fahrländer C Normand A-C Waser M Reboux G. High levels of grass pollen inside European dairy farms: a role for the allergy-protective effects of environment? Allergy. (2009) 64:1068–73. 10.1111/j.1398-9995.2009.01958.x 19220219

[72] Elieh Ali Komi D Shafaghat F Zwiener RD. Immunology of bee venom. Clini Rev Allergy Immunol. (2018) 54:386–96. 10.1007/s12016-017-8597-4 28105558

[73] Bilò MB Antonicelli L Bonifazi F. Honeybee venom immunotherapy: certainties and pitfalls. Immunotherapy. (2012) 4:1153–66. 10.2217/imt.12.113 23194365

[74] Müller UR. Insect venoms. In: Ring J, editor. Chemical Immunology and Allergy (Basel: KARGER). p. 141–156. 10.1159/000315948 20519887

[75] Hosono H Aoki J Nagai Y Bandoh K Ishida M Taguchi R. Phosphatidylserine-specific phospholipase A1 stimulates histamine release from rat peritoneal mast cells through production of 2-acyl-1-lysophosphatidylserine. J Biol Chem. (2001) 276:29664–70. 10.1074/jbc.M104597200 11395520

[76] Murakami M Hara N Kudo I Inoue K. Triggering of degranulation in mast cells by exogenous type II phospholipase A2. J Immunol. (1993) 151:5675. 8228255

[77] Dudler T Machado DC Kolbe L Annand RR Rhodes N Gelb MH. A link between catalytic activity, IgE-independent mast cell activation, and allergenicity of bee venom phospholipase A2. J Immunol. (1995) 155:2605. 7544378

[78] Palm NW Rosenstein RK Yu S Schenten DD Florsheim E Medzhitov R. Bee venom phospholipase A2 induces a primary type 2 response that Is dependent on the receptor ST2 and confers protective immunity. Immunity. (2013) 39:976–85. 10.1016/j.immuni.2013.10.006 24210353 PMC3852615

[79] Bourgeois EA Subramaniam S Cheng T-Y De Jong A Layre E Ly D. Bee venom processes human skin lipids for presentation by CD1a. J Exp Med. (2015) 212:149–63. 10.1084/jem.20141505 25584012 PMC4322046

[80] Jarrett R Salio M Lloyd-Lavery A Subramaniam S Bourgeois E Archer C. Filaggrin inhibits generation of CD1a neolipid antigens by house dust mite–derived phospholipase. Sci Transl Med. (2016) 8:325ra18. 10.1126/scitranslmed.aad6833 26865566 PMC4872823

[81] Dudeck A Dudeck J Scholten J Petzold A Surianarayanan S Köhler A. Mast cells are key promoters of contact allergy that mediate the adjuvant effects of haptens. Immunity. (2011) 34:973–84. 10.1016/j.immuni.2011.03.028 21703544

[82] Dudeck J Medyukhina A Fröbel J Svensson C-M Kotrba J Gerlach M. Mast cells acquire MHCII from dendritic cells during skin inflammation. J Exp Med. (2017) 214:3791–811. 10.1084/jem.20160783 29084819 PMC5716026

[83] Kato N Nakanishi M Hirashima N. Transbilayer asymmetry of phospholipids in the plasma membrane regulates exocytotic release in mast cells. Biochemistry. (2002) 41:8068–74. 10.1021/bi016022v 12069598

[84] Hammond GRV. Elimination of plasma membrane phosphatidylinositol (4,5)-bisphosphate is required for exocytosis from mast cells. J Cell Sci. (2006) 119:2084–94. 10.1242/jcs.02912 16687737

[85] Martin S Pombo I Poncet P David B Arock M Blank U. Immunologic stimulation of mast cells leads to the reversible exposure of phosphatidylserine in the absence of apoptosis. Int Arch Allergy Immunol. (2000) 123:249–58. 10.1159/000024451 11112862

[86] Suzuki J Fujii T Imao T Ishihara K Kuba H Nagata S. Calcium-dependent phospholipid scramblase activity of TMEM16 protein family members. J Biol Chem. (2013) 288:13305–16. 10.1074/jbc.M113.457937 23532839 PMC3650369

[87] SmrŽ D Dráberová L Dráber P. Non-apoptotic phosphatidylserine externalization Induced by engagement of glycosylphosphatidylinositol-anchored proteins. J Biol Chem. (2007) 282:10487–97. 10.1074/jbc.M611090200 17284440

[88] Martin TW Lagunoff D. Interactions of lysophospholipids and mast cells. Nature. (1979) 279:250–2. 10.1038/279250a0 86956

[89] Nakahashi-Oda C Tahara-Hanaoka S Shoji M Okoshi Y Nakano-Yokomizo T Ohkohchi N. Apoptotic cells suppress mast cell inflammatory responses via the CD300a immunoreceptor. J Exp Med. (2012) 209:1493–503. 10.1084/jem.20120096 22826299 PMC3409498

[90] Guo N Her GR Reinhold VN Brennan MJ Siraganian RP Ginsburg V. Monoclonal antibody AA4, which inhibits binding of IgE to high affinity receptors on rat basophilic leukemia cells, binds to novel &-galactosyl derivativesof ganglioside GDlb. J Biol Chem. (1989)13267–72. 2526814

[91] Mazucato VM Silveirae Souza AMM Nicoletti LM Jamur MC Oliver C. GD1b-derived gangliosides modulate FcεRI endocytosis in mast cells. J Histochem Cytochem. (2011) 59:428–40. 10.1369/0022155411400868 21411813 PMC3201145

[92] Filho EGF da Silva EZM Zanotto CZ Oliver C Jamur MC. Cross-linking mast cell specific gangliosides stimulates the release of newly formed lipid mediators and newly synthesized cytokines. Med Inflamm. (2016) 2016:1–10. 10.1155/2016/9160540 27578923 PMC4992799

[93] Pomorski TG Menon AK. Lipid somersaults: uncovering the mechanisms of protein-mediated lipid flipping. Progr Lipid Res. (2016) 64:69–84. 10.1016/j.plipres.2016.08.003 27528189 PMC5127727

[94] Saito K Fujimura-Kamada K Furuta N Kato U Umeda M Tanaka K. Cdc50p, a protein required for polarized growth, associates with the Drs2p P-type ATPase implicated in phospholipid translocation in *saccharomyces cerevisiae*. Mol Biol Cell. (2004) 15:3418–32. 10.1091/mbc.e03-11-0829 15090616 PMC452594

[95] Folmer DE Elferink RPJO Paulusma CC. P4 ATPases - lipid flippases and their role in disease. Biochim Biophys Acta. (2009) 1791:628–35. 10.1016/j.bbalip.2009.02.008 19254779

[96] Baldridge RD Graham TR. Identification of residues defining phospholipid flippase substrate specificity of type IV P-type ATPases. Proc Natl Acad Sci USA. (2012) 109:E290–8. 10.1073/pnas.1115725109 22308393 PMC3277569

[97] Panatala R Hennrich H Holthuis JCM. Inner workings and biological impact of phospholipid flippases. J Cell Sci. (2015) 128:2021–32. 10.1242/jcs.102715 25918123

[98] Cole SPC. Multidrug resistance protein 1 (MRP1, ABCC1), a “multitasking” ATP-binding cassette (ABC) transporter. J Biol Chem. (2014) 289:30880–8. 10.1074/jbc.R114.609248 25281745 PMC4223294

[99] Leier I Jedlitschky G Buchholz U Cole SP Deeley RG Keppler D. The MRP gene encodes an ATP-dependent export pump for leukotriene C4 and structurally related conjugates. J Biol Chem. (1994) 269:27807–10. 10.1097/00001813-199409001-00004 7961706

[100] Evers R Cnubben NH Wijnholds J van Deemter L van Bladeren PJ Borst P. Transport of glutathione prostaglandin A conjugates by the multidrug resistance protein 1. FEBS Lett. (1997) 419:112–6. 10.1016/S0014-5793(97)01442-7 9426231

[101] Brechbuhl HM Min E Kariya C Frederick B Raben D Day BJ. Select cyclopentenone prostaglandins trigger glutathione efflux and the role of ABCG2 transport. Free Radic Biol Med. (2009) 47:722–30. 10.1016/j.freeradbiomed.2009.06.005 19520157 PMC2730198

[102] Mitra P Oskeritzian CA Payne SG Beaven MA Milstien S Spiegel S. Role of ABCC1 in export of sphingosine-1-phosphate from mast cells. Proc Natl Acad Sci USA. (2006) 103:16394–9. 10.1073/pnas.0603734103 17050692 PMC1637593

[103] Quazi F Molday RS. Lipid transport by mammalian ABC proteins. Essays Biochem. (2011) 50:265–90. 10.1042/bse0500265 21967062

[104] Sahu SK Gummadi SN Manoj N Aradhyam GK. Phospholipid scramblases: an overview. Arch Biochem Biophys. (2007) 462:103–14. 10.1016/j.abb.2007.04.002 17481571

[105] Amir-Moazami O Alexia C Charles N Launay P Monteiro RC Benhamou M. Phospholipid scramblase 1 modulates a selected set of IgE receptor-mediated mast cell responses through LAT-dependent pathway. J Biol Chem. (2008) 283:25514–23. 10.1074/jbc.M705320200 18579528

[106] Zhou Q Zhao J Stout JG Luhm RA Wiedmer T Sims PJ. Molecular cloning of human plasma membrane phospholipid scramblase: a protein mediating transbilayer movement of plasma membrane phospholipids. J Biol Chem. (1997) 272:18240–4. 10.1074/jbc.272.29.18240 9218461

[107] Zhou Q. Normal hemostasis but defective hematopoietic response to growth factors in mice deficient in phospholipid scramblase 1. Blood. (2002) 99:4030–8. 10.1182/blood-2001-12-0271 12010804

[108] Wiedmer T Zhao J Nanjundan M Sims PJ. Palmitoylation of phospholipid scramblase 1 controls its distribution between nucleus and plasma membrane. Biochemistry. (2003) 42:1227–33. 10.1021/bi026679w 12564925

[109] Gilfillan AM Tkaczyk C. Integrated signalling pathways for mast-cell activation. Nat Rev Immunol. (2006) 6:218–30. 10.1038/nri1782 16470226

[110] Cui W Li S-Y Du J-F Zhu Z-M An P. Silencing phospholipid scramblase 1 expression by RNA interference in colorectal cancer and metastatic liver cancer. Hepatobil Pancr Dis Int. (2012) 11:393–400. 10.1016/S1499-3872(12)60197-0 22893466

[111] Kantari C Pederzoli-Ribeil M Amir-Moazami O Gausson-Dorey V Moura IC Lecomte M-C. Proteinase 3, the Wegener autoantigen, is externalized during neutrophil apoptosis: evidence for a functional association with phospholipid scramblase 1 and interference with macrophage phagocytosis. Blood. (2007) 110:4086–95. 10.1182/blood-2007-03-080457 17712045

[112] Huang Y Zhao Q Zhou C-X Gu Z-M Li D Xu H-Z. Antileukemic roles of human phospholipid scramblase 1 gene, evidence from inducible PLSCR1-expressing leukemic cells. Oncogene. (2006) 25:6618–27. 10.1038/sj.onc.1209677 16702944

[113] Ben-Efraim I Zhou Q Wiedmer T Gerace L Sims PJ. Phospholipid scramblase 1 is imported into the nucleus by a receptor-mediated pathway and interacts with DNA. Biochemistry. (2004) 43:3518–26. 10.1021/bi0356911 15035622

[114] Zhou Q Ben-Efraim I Bigcas J-L Junqueira D Wiedmer T Sims PJ. Phospholipid scramblase 1 binds to the promoter region of the inositol 1,4,5-triphosphate receptor type 1 gene to enhance its expression. J Biol Chem. (2005) 280:35062–8. 10.1074/jbc.M504821200 16091359

[115] Sun J Nanjundan M Pike LJ Wiedmer T Sims PJ. Plasma membrane phospholipid scramblase 1 is enriched in lipid rafts and interacts with the epidermal growth factor receptor. Biochemistry. (2002) 41:6338–45. 10.1021/bi025610l 12009895

[116] Nanjundan M Sun J Zhao J Zhou Q Sims PJ Wiedmer T. Plasma membrane phospholipid scramblase 1 promotes EGF-dependent activation of c-Src through the epidermal growth factor receptor. J Biol Chem. (2003) 278:37413–8. 10.1074/jbc.M306182200 12871937

[117] Pastorelli C Veiga J Charles N Voignier E Moussu H Monteiro RC. Phospholipid scramblase, a new effector of FcεRI signaling in mast cells. Mol Immunol. (2002) 38:1235–8. 10.1016/S0161-5890(02)00069-X 12217389

[118] Kassas A Moura IC Yamashita Y Scheffel J Guérin-Marchand C Blank U. Regulation of the tyrosine phosphorylation of phospholipid scramblase 1 in mast cells that are stimulated through the high-affinity IgE receptor. PLoS ONE. (2014) 9:e109800. 10.1371/journal.pone.0109800 25289695 PMC4188579

[119] Kassas-Guediri A Coudrat J Pacreau E Launay P Monteiro RC Blank U. Phospholipid scramblase 1 amplifies anaphylactic reactions *in vivo*. PLoS ONE. (2017) 12:e0173815. 10.1371/journal.pone.0173815 28282470 PMC5345872

[120] Dichlberger A Kovanen PT Schneider WJ. Mast cells: from lipid droplets to lipid mediators. Clini Sci. (2013) 125:121–30. 10.1042/CS20120602 23577635 PMC3631086

[121] Talukder AH Bao M Kim TW Facchinetti V Hanabuchi S Bover L. Phospholipid Scramblase 1 regulates Toll-like receptor 9-mediated type I interferon production in plasmacytoid dendritic cells. Cell Res. (2012) 22:1129–39. 10.1038/cr.2012.45 22453241 PMC3391020

[122] Tsai M-H Lee C-K. STAT3 cooperates with phospholipid scramblase 2 to suppress type I interferon response. Front Immunol. (2018) 9:1886. 10.3389/fimmu.2018.01886 30158934 PMC6104169

[123] Mutch DM O'Maille G Wikoff WR Wiedmer T Sims PJ Siuzdak G. Mobilization of pro-inflammatory lipids in obese Plscr3-deficient mice. Genome Biol. (2007) 8:R38. 10.1186/gb-2007-8-3-r38 17355638 PMC1868938

